# Repeal of Subminimum Wages and Social Determinants of Health Among People With Disabilities

**DOI:** 10.1001/jamahealthforum.2024.4034

**Published:** 2024-11-15

**Authors:** Mihir Kakara, Elizabeth F. Bair, Atheendar S. Venkataramani

**Affiliations:** 1 Department of Neurology, New York University Grossman School of Medicine, New York; 2 Leonard Davis Institute of Health Economics, University of Pennsylvania, Philadelphia; 3 Department of Medical Ethics and Health Policy, Perelman School of Medicine, University of Pennsylvania, Philadelphia

## Abstract

**Importance:**

People with disabilities experience pervasive health disparities driven by adverse social determinants of health, such as unemployment. Section 14(c) of the 1938 Fair Labor Standards Act has been a controversial policy that allows people with disabilities to be paid below the prevailing minimum wage, but its impact on employment remains unknown despite ongoing national debates about its repeal.

**Objective:**

To estimate whether state-level repeal of Section 14(c) was associated with employment-related outcomes for people with cognitive disability.

**Design, Setting, and Participants:**

This quasi-experimental, synthetic difference-in-differences study used individual-level data from the 2010-2019 American Community Surveys. Outcomes before and after subminimum wage law repeal in 2 states (New Hampshire and Maryland) that repealed Section 14(c) were compared with a synthetic group of control states that did not implement repeal. Individuals aged 18 to 45 years who reported having a cognitive disability were included. Data were analyzed from May 2023 to May 2024.

**Exposure:**

Repeal of Section 14(c) in New Hampshire (2015) and Maryland (2016).

**Main Outcomes and Measures:**

Primary outcomes were labor force participation and employment rates. Secondary outcomes were annual wages, annual hours worked, hourly wages, and proportion earning above state minimum wage among employed individuals.

**Results:**

The sample included 450 838 individuals. Of these, 253 157 (55.7%) were male, and the mean (SD) age was 31.3 (8.4) years. In state-specific analyses, New Hampshire’s labor force participation and employment had a statistically significant increase by 5.2 percentage points (β = 0.05; 95% CI, 0-0.10; *P* = .04) and 7 percentage points (β = 0.07; 95% CI, 0.01-0.13; *P* = .03), respectively, following Section 14(c) repeal. Labor force participation and employment both increased in Maryland, although estimates were not statistically significant. Pooling both states, Section 14(c) repeal was associated with a statistically significant 4.7–percentage point (β = 0.05; 95% CI, 0.01-0.08; *P* = .01) increase in labor force participation and a nonsignificant 4.3–percentage point (β = 0.04; 95% CI, 0-0.09; *P* = .07) increase in employment.

**Conclusions and Relevance:**

In this study, repeal of Section 14(c), a policy allowing subminimum wages for people with disabilities, led to increases in labor force participation, though with heterogeneity at the state level. These findings suggest the importance of state-level factors in shaping program effects, especially as national-level Section 14(c) repeal is being debated.

## Key Points

**Question:**

Does repeal of an 85-year-old law—Section 14(c) of the Fair Labor Standards Act—that allows for people with cognitive disabilities to be paid subminimum wages affect social determinants of health in this marginalized population?

**Findings:**

In this difference-in-differences study including 450 838 individuals from 2 states (New Hampshire and Maryland) that repealed Section 14(c), repeal was associated with a statistically significant increase in employment-related outcomes for people with cognitive disabilities in New Hampshire but not Maryland.

**Meaning:**

In this study, repeal of Section 14(c) led to improved employment-related outcomes in people with cognitive disabilities, although effects varied by state.

## Teaser

This difference-in-differences study estimates whether state-level repeal of Section 14(c) of the Fair Labor Standards Act was associated with employment-related outcomes for people with cognitive disability.

## Introduction

People with disabilities face significant barriers to accessing quality health care and experience poor health outcomes. Compared with people without disabilities, people with disabilities are less likely to receive preventive services,^1,2,3^ have higher rates of chronic health conditions,^3^ and have overall poorer self-reported health^3,4^ and mental health.^4,5^ People with disabilities are more likely to be unemployed,^6^ poor,^7^ and food insecure.^8^ These socioeconomic factors are critical drivers of health among people with disabilities: in a 2018 study,^9^ 32% of mental health disparities among working-age people with disabilities were explained by employment and financial hardship and were found to be more impactful than health behaviors. Other studies have linked unemployment and lower levels of education with greater psychological distress.^10,11^ These and other findings suggest that policies that target socioeconomic outcomes among people with disabilities may be important levers to improving health in this population.^12^

Policies that affect employment among people with disabilities may be particularly important given the importance of work for social integration and financial stability. However, barriers to employment and economic mobility for people with disabilities are pervasive. In addition to lack of supports and accommodations, discriminatory attitudes about productivity limit economic opportunities among people with disabilities.^13,14^ These attitudes have informed policymaking, as in the case of Section 14(c) of the Fair Labor Standard Act of 1938, which allows employers (who hold certificates issued by the US Department of Labor) to pay people with disabilities below the federal minimum wage. Workers employed in Section 14(c) jobs generally have intellectual and developmental disabilities (IDDs) and work in facility-based programs with controlled work environments. Work in these sheltered workshops, which ideally allow people with disabilities to acquire skills and services that would move them into more general work settings, typically involve repetitive activities, such as folding cardboard boxes, packaging products, and sorting recycling materials.

Section 14(c) is controversial. Proponents argue that repealing Section 14(c) would disincentivize employers from hiring people with disabilities, thereby decreasing their economic opportunities.^15^ Opponents of Section 14(c) cite potentially discriminatory and segregatory practices and lack of worker protections^16^ and argue that, with more investment in training, people with disabilities can be placed in integrated employment with equal wages. In this context, several states have repealed Section 14(c) in recent years, and the policy was recently scrutinized in a Government Accountability Office report finding significant federal labor law violations and $15 million in unpaid back wages by Section 14(c) employers.^17^ Further, a federal bill was introduced in Congress in 2021 to phase out all Section 14(c) jobs nationwide.^18^ Despite the long history of Section 14(c) legislation and contemporary debates about its continued appropriateness, little is known about the effects of repealing Section 14(c)—or the effects of policies targeting employment among people with disabilities more generally—on economic outcomes among people with disabilities. We assess the effects of state-level Section 14(c) repeals on key social determinants of health—labor force participation, employment, and earnings—using a quasi-experimental research design.

## Methods

### Data Sources and Study Population

We used survey data from the Integrated Public Use Microdata Series (IPUMS) US Census Bureau’s American Community Survey (ACS).^19^ The ACS is an annual, cross-sectional survey of 1% of US households, collecting information on social, economic, housing, and demographic characteristics. Race and ethnicity data were self-reported by participants in the ACS using a combination of checkboxes and write-in responses. We examined the period of 2010 to 2019, which begins after the Great Recession and before the COVID-19 pandemic, the latter of which introduced data collection challenges in the ACS and resulted in historic breaks in labor market outcomes among people with disabilities.^20^ Institutional review board review was not required given use of public deidentified data. This study followed the Strengthening the Reporting of Observational Studies in Epidemiology (STROBE) reporting guideline for cross-sectional studies.

We focused on individuals with IDD, who comprise most workers (90%) in Section 14(c) jobs.^17^ However, no large-scale data source has consistently identified people with IDD at the state level. To distinguish this population in our study, we focused on adults aged 18 to 45 years answering yes to the ACS question, “Because of a physical, mental, or emotional problem, do you have serious difficulty remembering, concentrating, or making decisions?” We chose this age range for 2 reasons. First, cognitive disabilities beyond the age of 45 years are more likely to include acquired and age-related causes, like stroke and dementia, which are less relevant to Section 14(c). Second, analysis of the National Health Interview Survey demonstrated that nearly 70% of individuals aged 18 to 45 years answering yes to the census-based measure of cognitive disability were reported to have intellectual disabilities, a proportion higher than for other age groups (eMethods in Supplement 1); this method of identifying the population of interest when direct markers are not available follows other work.^21^

### Outcomes

Our primary outcomes were labor force participation and employment. Labor force participation is a binary measure denoting whether the individual was working or actively looking for work in the 4 weeks prior to survey. Employment is a binary variable denoting whether the individual was employed at the time of survey. We also examined annual earned wages, total hours worked annually, and hourly wages—all calculated among employed workers reporting annual wages more than zero dollars—as secondary outcomes. Total hours worked was calculated by multiplying hours worked per week and weeks worked in the year. Annual earned wages were respondents’ total pretax wage, salary, commission, cash bonus, tip, and other income received as an employee for the previous 12 months. Hourly wages were calculated by dividing annual wages and total hours worked. All wages were adjusted for inflation (2019 US dollars).

### Exposure

We considered those living in 2 states that independently repealed Section 14(c) during our study period, New Hampshire (in 2015) and Maryland (in 2016), as exposed.

New Hampshire passed its bill prohibiting employers from employing individuals with disabilities at an hourly rate lower than the federal minimum wage, except for practical experience or training programs and family businesses.^22^ Maryland passed its bill with stipulations that, as of October 1, 2016, no work center should pay employees with disabilities a subminimum wage unless it was authorized to do so before this date. Indicating a phase-out process over 4 years, the bill stated that as of October 1, 2020, no centers should pay a subminimum wage under any circumstances.^23^

Although other states eliminated Section 14(c) in recent years (Alaska in 2018, Oregon in 2019, and Washington, Colorado, California, and Delaware in 2021), we did not include these repeals in our analyses because of their temporal proximity to the COVID-19 pandemic^24^ and the limited availability of posttreatment years of data. We also excluded Vermont, which eliminated Section 14(c) in 2002, and Alaska and Oregon, which eliminated Section 14(c) in 2018 and 2019, respectively, from the sample as they would not be pure controls.

### Statistical Analysis

We used synthetic difference-in-differences (DID)^25^ to compare changes in primary outcomes before and after Section 14(c) repeal in states that repealed the policy vs the same changes in states that did not. Synthetic DID combines the advantages of DID and synthetic control methods. The DID component of synthetic DID allows the preintervention period to have different outcome levels between the treatment and the control units. The synthetic control component of synthetic DID reweights all control units in the preintervention period to create a single matched synthetic unit that imposes parallel trends by construction, addressing a key assumption of DID required to support causal inference. Synthetic DID differs from traditional DID in that the control group is generated using unit-specific and time-specific weights that are calculated to generate comparisons that follow the same trends in outcomes (not necessarily levels) in the preperiod. The unit weights impose parallel trends and time weights improve precision by reducing outsize effects of time periods that are highly different relative to posttreatment periods. Synthetic DID differs from traditional synthetic control methods in its use of unit-fixed effects and time-specific weights. Unit-fixed effects (in this case, state-fixed effects) improve robustness by adjusting for unobserved state-specific characteristics of control states. Additionally, synthetic DID does not impose equal levels of outcomes in the preperiod as synthetic control does. The synthetic DID estimator has been used in other policy evaluation studies,^26^ and simulations suggest efficiency that weakly dominates DID or synthetic controls alone.^25^

To facilitate computation, we collapsed our individual-level ACS data to the state-year level, using ACS sampling weights to compute averages. We conducted state-specific analyses considering each treated state as an individual unit, where the other treated state was excluded from the control group. We also conducted analyses pooling both states using a staggered adoption design for synthetic DID.^25^ For inference, we used permutation testing to calculate SEs, a conservative procedure which is appropriate given the small number of treated units.

### Sensitivity Analysis

First, we used falsification tests to verify whether other policy changes that occurred during Section 14(c) may confound our estimates. We compared primary outcomes among people with no cognitive disability between treated states and synthetic controls. We also compared outcomes among people with noncognitive disabilities between treated states and synthetic controls. We expected null findings in both falsification checks, as Section 14(c) does not explicitly cover these groups of workers and the workforce with IDD is small enough that we would not expect their entry into the labor market to displace other workers. Second, we also repeated state-specific and pooled analyses for all outcomes using a standard synthetic control design.

In analyses of annual and hourly wages and proportion above state minimum wage, we included the year-specific state minimum wage as a time-varying covariate in the synthetic DID estimation. Exact tests were used to calculate *P* values. All hypotheses were 2-tailed, with *P* < .05 considered statistically significant. Stata/MP version 18.0 (StataCorp) was used for analyses. Data were analyzed from May 2023 to May 2024.

## Results

### Sample Characteristics

The sample included 450 838 people with cognitive disabilities aged 18 to 45 years in 47 states and the District of Columbia between 2010 and 2019 (weighted, 3.9% of all those aged 18 to 45 years in the sample states). Of these, 253 157 (55.7%) were male, and the mean (SD) age was 31.3 (8.4) years. Table 1 provides characteristics of the population with and without cognitive disability in the common pretreatment period of 2010 to 2014 across the 2 treated states (New Hampshire and Maryland) and the control states. A higher proportion of people with cognitive disability in the treated states vs the control states were in the labor force (adjusted percentage, 43.8% [95% CI, 43.5-44.2] in New Hampshire, 43.1% [95% CI, 42.9-43.3] in Maryland, and 38.2% [95% CI, 38.2-38.2] in control states) and employed (adjusted percentage, 31.6% [95% CI, 31.3-31.9] in New Hampshire, 31.1% [95% CI, 30.9-31.2] in Maryland, and 26.9% [95% CI, 26.9-26.9] in control states). Compared with people with no cognitive disability, a higher proportion of those with cognitive disabilities were not in the labor force and were unemployed in both the treated and control states. People with cognitive disabilities worked fewer hours and had lower annual and hourly wages. A lower proportion of people with cognitive disabilities completed any college education and received insurance other than Medicaid or Medicare.

**Table 1.** Baseline Characteristics of People Aged 18 to 45 Years With and Without Cognitive Disabilities Included in the Estimation Sample, 2010 to 2014^a^

Characteristic	No. (%)					
	New Hampshire		Maryland		All other states (except Vermont, Maryland, New Hampshire, Alaska, and Oregon)^b^	
	No cognitive disability	Cognitive disability	No cognitive disability	Cognitive disability	No cognitive disability	Cognitive disability
Total, No.	20 188	880	96 900	3448	4 862 029	210 418
Age, mean (SD), y	31.6 (8.5)	30.6 (8.5)	31.5 (8.2)	30.8 (8.4)	31.2 (8.2)	31.3 (8.4)
Sex						
Female	10 273 (50.3)	394 (42.7)	49 676 (50.9)	1476 (42.7)	2 440 766 (49.9)	92 427 (44.5)
Male	9915 (49.7)	486 (57.3)	47 224 (49.1)	1972 (57.3)	2 421 263 (50.1)	117 991 (55.5)
Race and ethnicity^c^						
American Indian or Alaska Native	43 (0.2)	3 (0.1)	268 (0.3)	20 (0.5)	53 695 (0.8)	3585 (1.4)
Asian	651 (3.4)	8 (0.9)	6993 (7.0)	91 (3.0)	305 808 (6.3)	4717 (2.2)
Black	251 (1.4)	15 (2.1)	25 479 (30.5)	1237 (37.7)	537 335 (12.9)	34 851 (17.4)
Hispanic^d^	551 (3.7)	30 (4.2)	8759 (11.4)	150 (4.6)	839 428 (20.1)	27 628 (14.4)
White	18 334 (89.5)	792 (88.9)	53 085 (48.5)	1817 (50.3)	3 020 957 (57.6)	132 215 (61.1)
Other race^e^	358 (1.8)	32 (3.6)	2316 (2.3)	133 (3.9)	104 806 (2.1)	7422 (3.6)
Insurance						
Medicaid	1131 (5.8)	429 (47.4)	9491 (10.4)	1853 (54.9)	526 740 (10.9)	108 660 (50.9)
Medicare	198 (1.1)	199 (22.2)	800 (0.9)	475 (14.6)	45 787 (0.9)	37 037 (17.2)
No insurance	3204 (17.5)	151 (19.3)	14 289 (17.3)	500 (13.4)	1 068 664 (24.2)	44 440 (20.9)
Any college education	12 277 (59.4)	237 (27.2)	59 735 (59.3)	909 (26.7)	2 703 619 (55.1)	52 151 (25.8)
In labor force^f^	16 615 (83.8)	367 (43.8)	79 245 (83.2)	1384 (43.1)	3 809 818 (79.7)	74 816 (38.2)
Employed^g^	15 549 (78.0)	267 (31.6)	72 692 (75.7)	992 (31.1)	3 441 603 (71.8)	53 063 (26.9)
Annual wages, mean (SD), $^h^	18 906.3 (20 716.9)	8600.3 (8775.1)	21 475.3 (21 819.8)	10 798.6 (11 907.3)	17 892.8 (20 702.5)	9632.2 (12 905.5)
Annual hours worked, mean (SD)^i^	1840.3 (729.6)	1388.6 (787.8)	1877.5 (704.2)	1427.8 (791.9)	1851.6 (732.1)	1439.3 (866.9)
Hourly wages, mean (SD), $^i^^,^^j^	9.9 (14.0)	5.9 (4.3)	11.1 (12.5)	6.9 (6.2)	9.4 (20.1)	7.1 (60.4)

^a^ The period of 2010 to 2014 was chosen as this was the common period for all listed states prior to any Section 14(c) repeal.

^b^ Vermont was excluded as it eliminated Section 14(c) in 2002. Alaska and Oregon were excluded as they eliminated Section 14(c) in 2018 and 2019, respectively, and would not be pure controls.

^c^ Race and ethnicity were self-reported in the American Community Survey.

^d^ Includes the following categories: Cuban, Mexican, Puerto Rican, Costa Rican, Guatemalan, Honduran, Nicaraguan, Panamanian, Salvadoran, Argentinian, Bolivian, Chilean, Colombian, Ecuadorian, Paraguayan, Peruvian, Uruguayan, Venezuelan, Spaniard, Dominican, and other Hispanic ethnicities not included in above categories.

^e^ The other race category includes all other races that are multiracial and not elsewhere classified.

^f^ Labor force participation denotes if an individual was working or actively looking for work in the 4 weeks prior to survey.

^g^ Employment denotes whether the individual was employed at the time of survey.

^h^ All dollar values were adjusted to 2019 US dollars.

^i^ Total hours worked was calculated by multiplying hours worked per week and weeks worked in the year.

^j^ Hourly earned income was calculated by dividing annual earned income by total hours worked in the year.

### Synthetic DID Estimates

**Figure 1.**
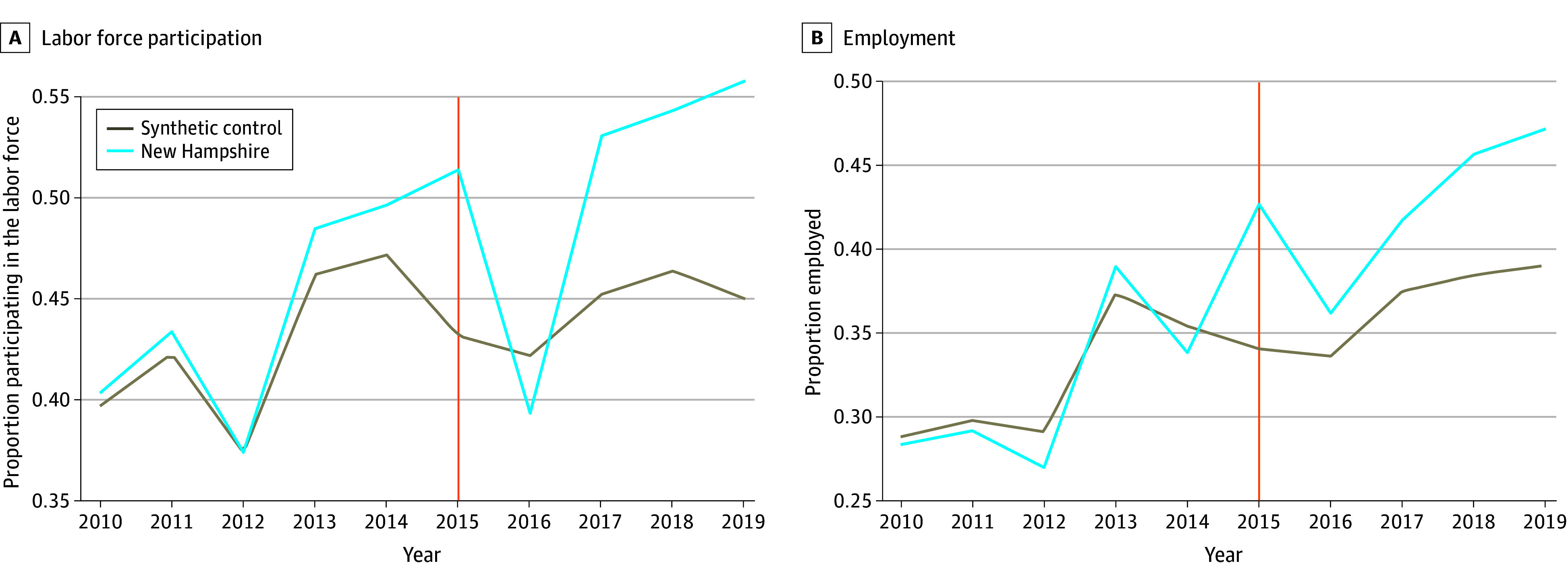
Primary Outcomes for New Hampshire Trends in labor force participation and employment rates in New Hampshire following repeal of Section 14(c) of the Fair Labor Standards Act in 2015 compared with synthetic control. The synthetic difference-in-differences estimator constructs a weighted average of all other states that did not repeal Section 14(c) and imposes a parallel trend in the period prior to the repeal so that the treated unit and synthetic control are similar to each other. The vertical line indicates when Section 14(c) was repealed.

In analyses focusing on New Hampshire relative to its synthetic control group (Table 2), both labor force participation and employment rates increased significantly by 5.2 percentage points (β = 0.05; 95% CI, 0-0.10; *P* = .04) (Figure 1A) and 7 percentage points (β = 0.07; 95% CI, 0.01-0.13; *P* = .03) (Figure 1B), respectively, following repeal. Unit (state) and time weights computed to generate synthetic DID control groups are presented in eTables 1 to 4 and eFigures 3 to 6 in Supplement 1. The estimates for labor force participation and employment represent a weighted 11.9% (4856 workers) and 21.9% (6434 workers) increase, respectively, relative to their prerepeal means. There were no significant changes in total hours worked, annual and hourly wages, or proportion earning above the state minimum wage in the treated population in New Hampshire following Section 14(c) repeal among those employed (eFigure 1 in Supplement 1).

**Table 2.** Synthetic Difference-in-Differences Estimates for New Hampshire and Maryland Following Section 14(c) Repeal Compared With Synthetic Control

Outcome	New Hampshire		Maryland		Pooled estimates	
	β (95% CI)	*P* value	β (95% CI)	*P* value	β (95% CI)	*P* value
Primary outcomes						
Labor force participation^a^	0.05 (0 to 0.10)	.04	0.04 (−0.02 to 0.10)	.20	0.05 (0.01 to 0.08)	.01
Employment^b^	0.07 (0.01 to 0.13)	.03	0.01 (−0.06 to 0.09)	.75	0.04 (0 to 0.09)	.07
Secondary outcomes						
Annual wages (logged)^c^	−0.04 (−0.30 to 0.22)	.76	−0.07 (−0.30 to 0.15)	.52	−0.06 (−0.22 to 0.11)	.50
Annual hours worked^d^	−48.12 (−189.10 to 92.86)	.50	−14.49 (−138.29 to 109.31)	.82	−33.17 (−118.91 to 52.56)	.45
Hourly wages (logged)^e^	0.04 (−0.14 to 0.22)	.65	0 (−0.14 to 0.14)	.95	0.02 (−0.10 to 0.14)	.73
Proportion earning above state minimum wage, %	9.26 (−1.45 to 19.98)	.09	−2.02 (−11.53 to 7.50)	.68	4.25 (−2.21 to 10.71)	.20

^a^ Labor force participation denotes if an individual was working or actively looking for work in the 4 weeks prior to survey.

^b^ Employment denotes whether the individual was employed at the time of survey.

^c^ All dollar values were adjusted to 2019 US dollars.

^d^ Total hours worked was calculated by multiplying hours worked per week and weeks worked in the year.

^e^ Hourly earned income was calculated by dividing annual earned income by total hours worked in the year.

**Figure 2.**
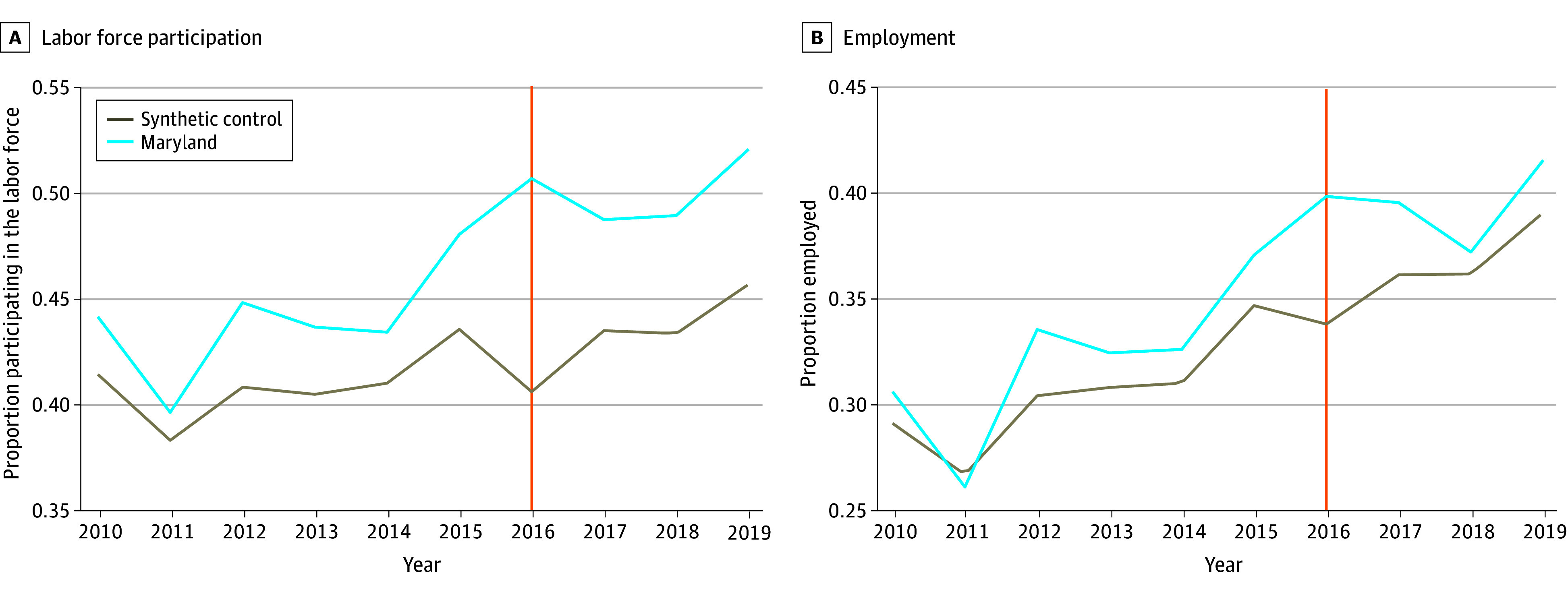
Primary Outcomes for Maryland Trends in labor force participation and employment rates in Maryland following repeal of Section 14(c) of the Fair Labor Standards Act in 2016 compared with synthetic control. The synthetic difference-in-differences estimator constructs a weighted average of all other states that did not repeal Section 14(c) and imposes a parallel trend in the period prior to the repeal so that the treated unit and synthetic control are similar to each other. The vertical line indicates when Section 14(c) was repealed.

In analyses focusing on Maryland (Table 2), there were smaller and nonsignificant changes in labor force participation and employment following the repeal of Section 14(c) in 2016 relative to its synthetic control group (Figure 2). Similarly, there were no significant changes in secondary outcomes following Section 14(c) repeal for employed individuals (eFigure 2 in Supplement 1).

In pooled analysis including both treated states (Table 2), following repeal, there was a statistically significant 4.7–percentage point (β = 0.05; 95% CI, 0.01-0.08; *P* = .01) increase in labor force participation among people with cognitive disabilities in the 2 states repealing Section 14(c) compared with its synthetic control. This is equivalent to a weighted 10.7% (24 575 workers) increase in labor force participation relative to the mean among both states before the repeal. Employment rates increased following Section 14(c) repeal by 4.3 percentage points (β = 0.04; 95% CI, 0-0.09; *P* = .07). There were no statistically significant changes in the secondary outcomes following repeal among employed individuals.

### Sensitivity Analyses

We found no differences in labor force participation or employment rates among people without cognitive disability in comparisons of both New Hampshire vs synthetic control and Maryland vs synthetic control (eTable 5 in Supplement 1). Similarly, there were no differences in labor force participation or employment rates among people with noncognitive disabilities in comparisons of both New Hampshire vs synthetic control and Maryland vs synthetic control. Estimates using standard synthetic control methods yielded substantively similar results (eTable 6 and eFigures 7 to 10 in Supplement 1).

## Discussion

In this quasi-experimental study evaluating the population-level effects of the repeal of Section 14(c), an 86-year-old legislation that allows employers to pay workers with disabilities lower than the federal minimum wage, on key social determinants of health, we found overall heterogenous effects on people with cognitive disabilities, with New Hampshire having statistically significant increases in both primary outcomes (labor force participation and employment) following repeal and Maryland with smaller but nonsignificant increases in labor force participation and employment. Pooled analyses combining both states showed a significant increase in labor force participation and similarly large but nonsignificant increase in employment rates among persons with cognitive disabilities following Section 14(c) repeal. Falsification tests examining effects among people with no cognitive disabilities and people with noncognitive disabilities yielded smaller, nonsignificant estimates, suggesting that our findings are likely not explained by any other state-specific employment policy. To our knowledge, this study is the first to assess the effects of repealing Section 14(c).

The results have several policy implications. First, the significant increase in overall labor force participation may reflect the inclusive nature of repealing Section 14(c), as it brings people with cognitive disabilities previously not connected with employment resources and training into the labor force (with a potential lag in subsequent active employment). Second, among the findings with no statistical significance, the 95% CIs of employment rate in pooled analyses (β = 0.04; 95% CI, 0-0.09) and Maryland’s labor force participation rate (β = 0.04; 95% CI, −0.02 to 0.10) seem to rule out any large negative effects of Section 14(c) repeal in these contexts. This is relevant, as negative effects are a concern often cited by proponents of Section 14(c).^15^ However, detrimental effects on subpopulations with IDD cannot be ruled out.

Third, the differential increases in labor market outcomes in New Hampshire compared with Maryland underscore the role of state-specific factors. One potential explanation is that New Hampshire provided significantly higher per-capita funding for integrated employment training specifically for people with IDD compared with Maryland in the years surrounding the repeal (eFigure 11 in Supplement 1), given prior work showing that better integrated employment funding improved labor market outcomes for individuals with IDD.^27^ However, there may be other potential drivers of differential program effects, including the types of industries employing workers with IDD, availability of other employment support and welfare programs, caregiving policies, and social norms. Our findings demonstrate the importance of identifying these factors for future research and policymaking.

Fourth, we note that New Hampshire did not have any Section 14(c) workers in 2015 at the time of repeal^28^ but still saw an increase in both labor force participation and employment rate. This suggests that policy effects may operate through other mechanisms besides raising the minimum wage—though the effect of raising the minimum wage itself can invite new workers into the labor force.^29^ For example, in addition to increased integrated employment funding increasing IDD agencies’ capacity to provide employment training, there is growing evidence that policies may have effects beyond their designed or material effects via signaling and affective pathways, especially for charged policy issues.^30^ In this case, media coverage and debates around Section 14(c) repeal might encourage or signal to families and individuals with IDD previously out of the labor force to apply for employment training. Exploration of these factors is outside this study’s scope and should be pursued in future work. Another potential explanation for the differences in employment outcomes between New Hampshire and Maryland is that Maryland’s bill specified a gradual phase-out plan for subminimum wage jobs over 4 years rather than an immediate elimination of all jobs. As a result, Maryland still had 1513 Section 14(c) workers as of April 2019.^28^ It is possible that because of this gradual phase-out, effects of the repeal, if any, would be more obvious after 2020. Finally, the lack of significant changes in the secondary outcomes likely reflect heterogeneity in either specific occupation types or other multilayered factors affecting wages that could arise from employment (eg, taxes, health insurance, transportation costs)—something that is outside the scope of this study.

### Limitations

Our study has limitations, all of which motivate future work. First, given data constraints, we cannot estimate the individual-level effects of Section 14(c) repeals. Although our findings suggest no obvious adverse population-level economic effects on people with cognitive disabilities, individual Section 14(c) workers, especially those with severe IDD, may become unemployed following employers’ inability to pay subminimum wages. Individual-level data on people transitioning out of Section 14(c) jobs should be collected. Second, our data cannot allow us to definitively identify intellectual and psychiatric disabilities, the groups that constitute almost all Section 14(c) workers and are known to be undercounted in most surveys.^31,32^ However, we could identify the population on which Section 14(c) is most likely to impact—an approach that has been previously used in policy evaluations where data availability has been an issue.^21^ Additionally, as shown previously, it is possible that the ACS disability question, based on its binary responses, and potentially question phrasing likely selects for relatively higher levels of intellectual disability compared with the National Health Interview Survey, which uses a 4-point Likert scale.^33^ Third, we only are able to evaluate treatment effects in 2 states, and examining policy effects in other states repealing Section 14(c) will be important for future work. Fourth, although synthetic DID methodology requires only 2 pretreatment time periods to estimate the synthetic control, our dataset had a relatively short pretreatment panel of 5 years. Finally, while we examine a key set of social determinants of health, data constraints render us unable to measure policy impacts on health directly. We hope our research stimulates further work examining whether equity in minimum wages among people with disabilities translates to health equity.

## Conclusions

Repeal of Section 14(c), an 85-year-old policy allowing subminimum wages for people with disabilities, had an overall increase in labor force participation, although with heterogeneity at the state level. These findings suggest the importance of state-level factors in shaping program effects, especially as national-level Section 14(c) repeal is being debated.

## Notes

Supplement 1.
eMethods.

eReferences.
**eFigure 1.** Secondary Outcomes for New Hampshire Using Synthetic Difference-in-Differences **eFigure 2.** Secondary Outcomes for Maryland Using Synthetic Difference-in-Differences **eTable 1.** Unit Weights for Synthetic Control in New Hampshire-Specific SDID Analyses **eFigure 3.** Unit Weights for Synthetic Control in New Hampshire-Specific SDID Analyses: Primary Outcomes (Graph) **eFigure 4.** Unit Weights for Synthetic Control in New Hampshire-Specific SDID Analyses: Secondary Outcomes (Graph) **eTable 2.** Unit Weights for Synthetic Control in Maryland-Specific SDID Analyses **eFigure 5.** Unit Weights for Synthetic Control in Maryland-Specific SDID Analyses: Primary Outcomes (Graph) **eFigure 6.** Unit Weights for Synthetic Control in Maryland-Specific SDID Analyses: Secondary Outcomes (Graph) **eTable 3.** Time Weights for Synthetic Control in New Hampshire-Specific SDID Analyses **eTable 4.** Time Weights for Synthetic Control in Maryland-Specific SDID Analyses **eTable 5.** Falsification Tests **eTable 6.** Estimates for New Hampshire, Maryland and Pooled Analyses Using Standard Synthetic Control Methodology **eFigure 7.** Primary Outcomes for New Hampshire Using Standard Synthetic Control Methodology **eFigure 8.** Secondary Outcomes for New Hampshire Using Standard Synthetic Control Methodology **eFigure 9.** Primary Outcomes for Maryland Using Standard Synthetic Control Methodology **eFigure 10.** Secondary Outcomes for Maryland Using Standard Synthetic Control Methodology **eFigure 11.** Per-Capita State Funding for Competitive Integrated Employment for Individuals With Intellectual Disabilities

Supplement 2. Data Sharing Statement
