# Supplementary material for: Repeal of Subminimum Wages and Social Determinants of Health Among People With Disabilities
Source: JAMA Health Forum. 2024 Nov 15;5(11):e244034. doi: 10.1001/jamahealthforum.2024.4034 (PMC11568457; doi:10.1001/jamahealthforum.2024.4034)
Supplement: Supplement 1. — eMethods. eReferences. eFigure 1. Secondary Outcomes for New Hampshire Using Synthetic Difference-in-Differences eFigure 2. Secondary Outcomes for Maryland Using Synthetic Difference-in-Differences eTable 1. Unit Weights for Synthetic Control in New Hampshire-Specific SDID Analyses eFigure 3. Unit Weights for Synthetic Control in New Hampshire-Specific SDID Analyses: Primary Outcomes (Graph) eFigure 4. Unit Weights for Synthetic Control in New Hampshire-Specific SDID Analyses: Secondary Outcomes (Graph) eTable 2. Unit Weights for Synthetic Control in Maryland-Specific SDID Analyses eFigure 5. Unit Weights for Synthetic Control in Maryland-Specific SDID Analyses: Primary Outcomes (Graph) eFigure 6. Unit Weights for Synthetic Control in Maryland-Specific SDID Analyses: Secondary Outcomes (Graph) eTable 3. Time Weights for Synthetic Control in New Hampshire-Specific SDID Analyses eTable 4. Time Weights for Synthetic Control in Maryland-Specific SDID Analyses eTable 5. Falsification Tests eTable 6. Estimates for New Hampshire, Maryland and Pooled Analyses Using Standard Synthetic Control Methodology eFigure 7. Primary Outcomes for New Hampshire Using Standard Synthetic Control Methodology eFigure 8. Secondary Outcomes for New Hampshire Using Standard Synthetic Control Methodology eFigure 9. Primary Outcomes for Maryland Using Standard Synthetic Control Methodology eFigure 10. Secondary Outcomes for Maryland Using Standard Synthetic Control Methodology eFigure 11. Per-Capita State Funding for Competitive Integrated Employment for Individuals With Intellectual Disabilities [file jamahealthforum-e244034-s001.pdf]

Supplemental Online Content

Kakara M, Bair EF, Venkataramani AS. Repeal of subminimum wages and social determinants of health among people with disabilities. *JAMA Health Forum.* 2024;5(11):e244034. doi:10.1001/jamahealthforum.2024.4034

eMethods.
eReferences.
eFigure 1. Secondary Outcomes for New Hampshire Using Synthetic Difference-in-Differences
eFigure 2. Secondary Outcomes for Maryland Using Synthetic Difference-in-Differences
eTable 1. Unit Weights for Synthetic Control in New Hampshire-Specific SDID Analyses
eFigure 3. Unit Weights for Synthetic Control in New Hampshire-Specific SDID Analyses: Primary Outcomes (Graph)
eFigure 4. Unit Weights for Synthetic Control in New Hampshire-Specific SDID Analyses: Secondary Outcomes (Graph)
eTable 2. Unit Weights for Synthetic Control in Maryland-Specific SDID Analyses
eFigure 5. Unit Weights for Synthetic Control in Maryland-Specific SDID Analyses: Primary Outcomes (Graph)
eFigure 6. Unit Weights for Synthetic Control in Maryland-Specific SDID Analyses: Secondary Outcomes (Graph)
eTable 3. Time Weights for Synthetic Control in New Hampshire-Specific SDID Analyses
eTable 4. Time Weights for Synthetic Control in Maryland-Specific SDID Analyses
eTable 5. Falsification Tests
eTable 6. Estimates for New Hampshire, Maryland and Pooled Analyses Using Standard Synthetic Control Methodology
eFigure 7. Primary Outcomes for New Hampshire Using Standard Synthetic Control Methodology
eFigure 8. Secondary Outcomes for New Hampshire Using Standard Synthetic Control Methodology
eFigure 9. Primary Outcomes for Maryland Using Standard Synthetic Control Methodology
eFigure 10. Secondary Outcomes for Maryland Using Standard Synthetic Control Methodology
eFigure 11. Per-Capita State Funding for Competitive Integrated Employment for Individuals With Intellectual Disabilities

This supplementary material has been provided by the authors to give readers additional information about their work.

© 2024 Kakara M et al. *JAMA Health Forum*.

eMethods.

Sample identification:
Since the ACS does not collect data on health conditions, we used another dataset, the National Health Interview Survey (NHIS),[1] to cross-check to what extent people with intellectual disability report themselves (or are reported by proxies) to have a cognitive disability in nationally representative surveys. The NHIS asks survey respondents if there is any activity limitation from a range of conditions, one of which is intellectual disability. It also asks all adult respondents to rate the "amount of difficulty remembering or concentrating" on a 4-point Likert scale, with 'no difficulty,' 'some difficulty,' 'a lot of difficulty,' and 'cannot do at all.' We find that adults aged 18-45 with cognitive difficulties (defined as those with any difficulty) are the age group with the most enriched fraction of adults with intellectual disabilities – 68.9% of people in this age group who have cognitive difficulty report having intellectual disability.

Race and Ethnicity: Both race and ethnicity were self-reported by participants in the American Community Survey. 'Other' race includes all other races that are multiracial and not elsewhere classified. Ethnicity included the following ethnicities: Cuban, Mexican, Puerto Rican, Costa Rican, Guatemalan, Honduran, Nicaraguan, Panamanian, Salvadoran, Argentinian, Bolivian, Chilean, Colombian, Ecuadorian, Paraguayan, Peruvian, Uruguayan, Venezuelan, Spaniard, Dominican and other Hispanic ethnicities not included in above categories.

Statistical analyses:
For implementing the synthetic difference-in-differences analyses, the Stata 'sdid' command developed by Clarke et al. was used.[2]

To calculate per capita funding for integrated employment training shown in eFigure 11 of Supplement, data was obtained from ThinkWork,[3] an online data hub that includes data from the National Survey of State IDD Agencies' Employment and Day Services. Per-capita funding was then calculated by divided total funding for integrated employment divded by number of people with IDD served.

© 2024 Kakara M et al. *JAMA Health Forum*.

eReferences.

1. Blewett L, Drew JAR, King ML, et al. IPUMS Health Surveys: National Health Interview Survey, Version 7.3 [dataset]. Minneapolis, MN: IPUMS, 2023. https://doi.org/10.18128/D070.V7.3

2. Clarke D, Pailañir D, Carleton Athey S, Imbens GW. Synthetic Difference-in-Differences Estimation. *SSRN Journal*. Published online 2023. doi:10.2139/ssrn.4346540

3. Statedata.info. (2024). State IDD Agencies. U.S. Total: Integrated employment funding. Retrieved 04/15/2024 from http://statedata.thinkwork.org/data/showchart/244424

© 2024 Kakara M et al. *JAMA Health Forum*.

eFigure 1. Secondary Outcomes for New Hampshire Using Synthetic Difference-in-Differences

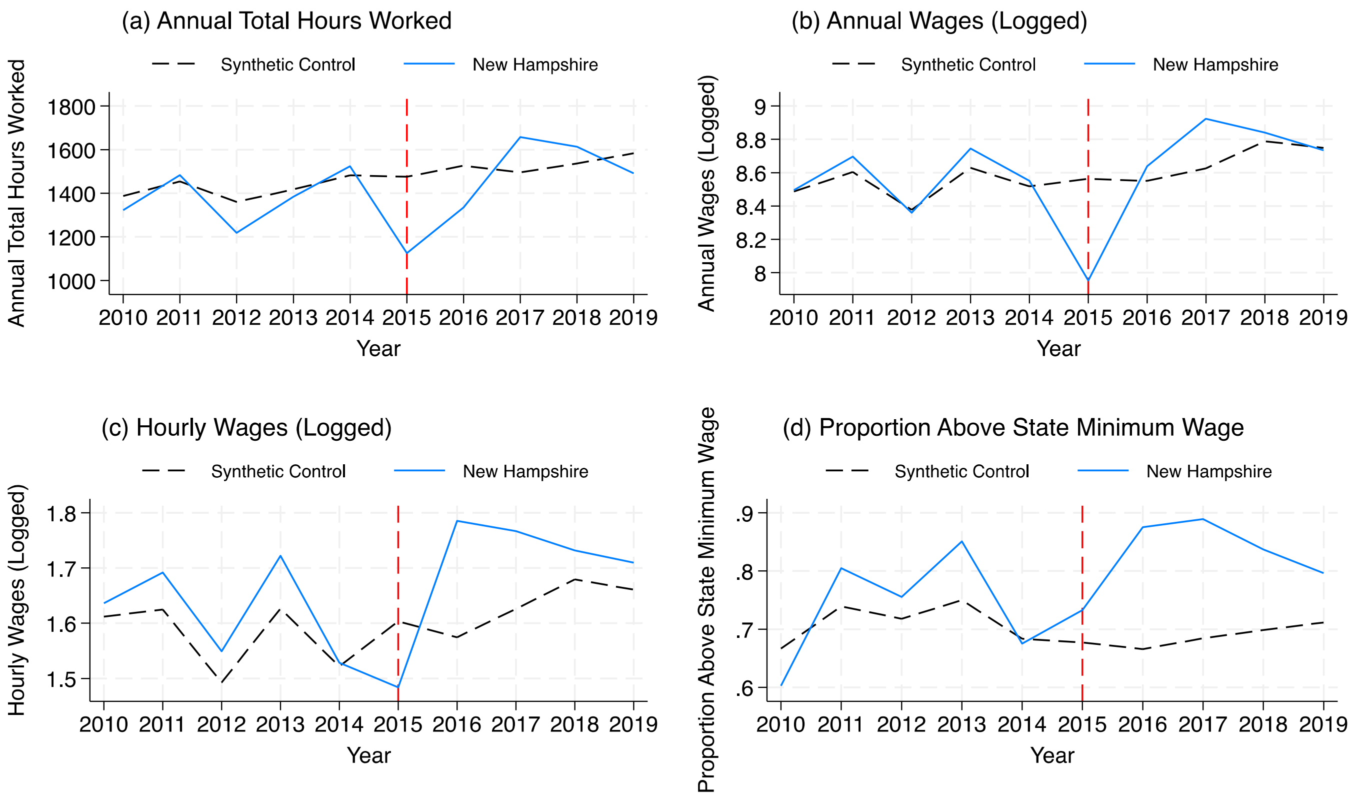

Trends in annual total hours worked, logged annual wages, logged hourly wages and proportion above state minimum wage in New Hampshire following repeal of Section 14(c) in 2015 as compared to synthetic control. The synthetic difference-in-differences estimator constructs a weighted average of all other states that did not repeal Section 14(c), and imposes a parallel trends in the period prior to the repeal so that treated unit and synthetic control are similar to each other.

© 2024 Kakara M et al. *JAMA Health Forum*.

eFigure 2. Secondary Outcomes for Maryland Using Synthetic Difference-in-Differences

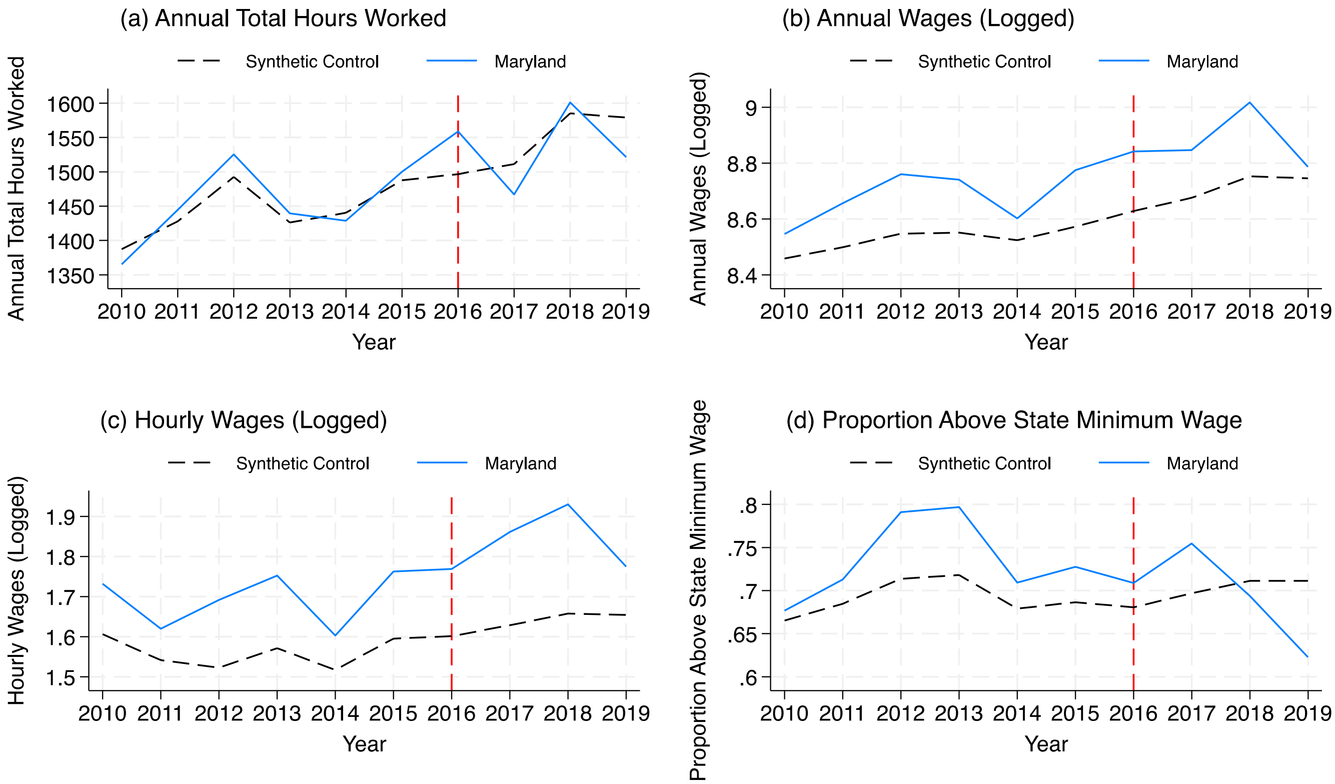

Trends in annual total hours worked, logged annual wages, logged hourly wages and proportion above state minimum wage in Maryland following repeal of Section 14(c) in 2016 as compared to synthetic control. The synthetic difference-in-differences estimator constructs a weighted average of all other states that did not repeal Section 14(c), and imposes a parallel trends in the period prior to the repeal so that treated unit and synthetic control are similar to each other.

© 2024 Kakara M et al. *JAMA Health Forum*.

eTable 1. Unit Weights for Synthetic Control in New Hampshire-Specific SDID Analyses

State	Labor Force Participation	Employment	Annual Total Hours Worked	Annual Wages	Hourly Wages	Proportion above minimum wage
Connecticut	.01521599	0	0	.00032776	.00305678	.00002281
Maine	.01466657	0	.09582836	.05290411	.01727579	.00002281
Massachusetts	.02986232	.03578003	0	.01402772	.03552363	.00002281
Rhode Island	.05619463	.03547903	.07030345	.07963938	.05859689	.0889371
Delaware	.02901803	.01953657	.03415781	.03259862	.03163121	.02180183
New Jersey	.01073151	.01714536	0	.00032776	.00176732	.00002281
New York	.01777306	.00115658	.00309363	.01369891	.02632222	.04378811
Pennsylvania	.02365767	.00792461	.03291814	.0267038	.02825558	.01939486
Illinois	.01988503	.01127124	.01967766	.01156721	.00573721	.00002281
Indiana	.0090269	.00986027	0	.02378073	.02935849	.00227103
Michigan	0	.01217746	0	.01924359	.0364352	.01979704
Ohio	.01402891	.00292229	0	.00871725	.01975476	.00002281
Wisconsin	.03431699	.02228143	.00716014	.00032776	.00056855	.00002281
Iowa	.01948935	.04590019	.0188674	.01436039	.00056855	.00002281
Kansas	.01536471	.03099138	.05382705	.04192116	.00056855	.00002281
Minnesota	.01781652	.01617115	.0372105	.03364632	.04070555	.18268736
Missouri	.01367461	.01050052	0	.00351912	.01359128	.00002281
Nebraska	.0018193	.02720752	0	.03318665	.00056855	.00002281
North Dakota	.17408484	.15722317	.06599782	.0600853	.03817038	.00002281
South Dakota	.02765276	.04739911	.12119627	.05317486	.05086105	.0483268
Virginia	.03575617	.04000165	0	.00219576	.01353497	.00002281
Alabama	.01433163	.03281063	0	.02183173	.01636653	.00002281
Arkansas	0	0	.06039175	.01109488	.00997344	.00002281
Florida	.00417425	0	.04428392	.02236629	.01772266	.00219002
Georgia	.01249981	.00240145	0	.02567628	.03593207	.04520277
Louisiana	.00630226	0	.00081841	.01728121	.02878467	.03132568
Mississippi	.01221127	0	0	.00032776	.00056855	.04407542
North Carolina	0	0	.05130013	.02832468	.02000742	.00002281
South Carolina	.04546511	.07778666	.03680433	.03419358	.05650322	.1365579
Texas	.01237787	.01888729	.03783879	.01505064	.02689281	.00362364
Kentucky	.03676953	0	.03172626	.01072121	.0093227	.00002281
Oklahoma	0	.00897475	.02278479	.02308712	.00056855	.00002281
Tennessee	.02201001	.03954215	0	.01602716	.00749927	.00002281
West Virginia	.0259718	.01221768	.01435612	.00032776	.00056855	.00002281
Arizona	0	0	0	.00378788	.03119764	.00601499
Colorado	.0106756	.02972743	.04471354	.02579562	.03400011	.02299503
Idaho	.00286815	.01447411	0	.00032776	.00056855	.00002281
Montana	.02925331	.02717578	0	.00032776	.04205038	.00002281
Nevada	.03672998	.00592925	.00520287	.01165502	.00056855	.00002281
New Mexico	.00868124	.07430364	.03772507	.01889871	.02548704	.09520836
Utah	0	0	0	.00279573	.0262153	.00002281
Wyoming	.01044598	.01899678	.02003092	.12350535	.08696157	.04885564
California	.01817667	.03414404	.02039431	.0219445	.01108007	.00002281
Washington	.03980153	.01755919	.01139058	.03804173	.01544759	.00002281
Hawaii	0	0	0	.00032776	.02453481	.13633043
District of Columbia	.07121815	.0341396	0	.00032776	.01832542	.00002281

© 2024 Kakara M et al. *JAMA Health Forum*.

eFigure 3. Unit Weights for Synthetic Control in New Hampshire-Specific SDID Analyses: Primary Outcomes (Graph)

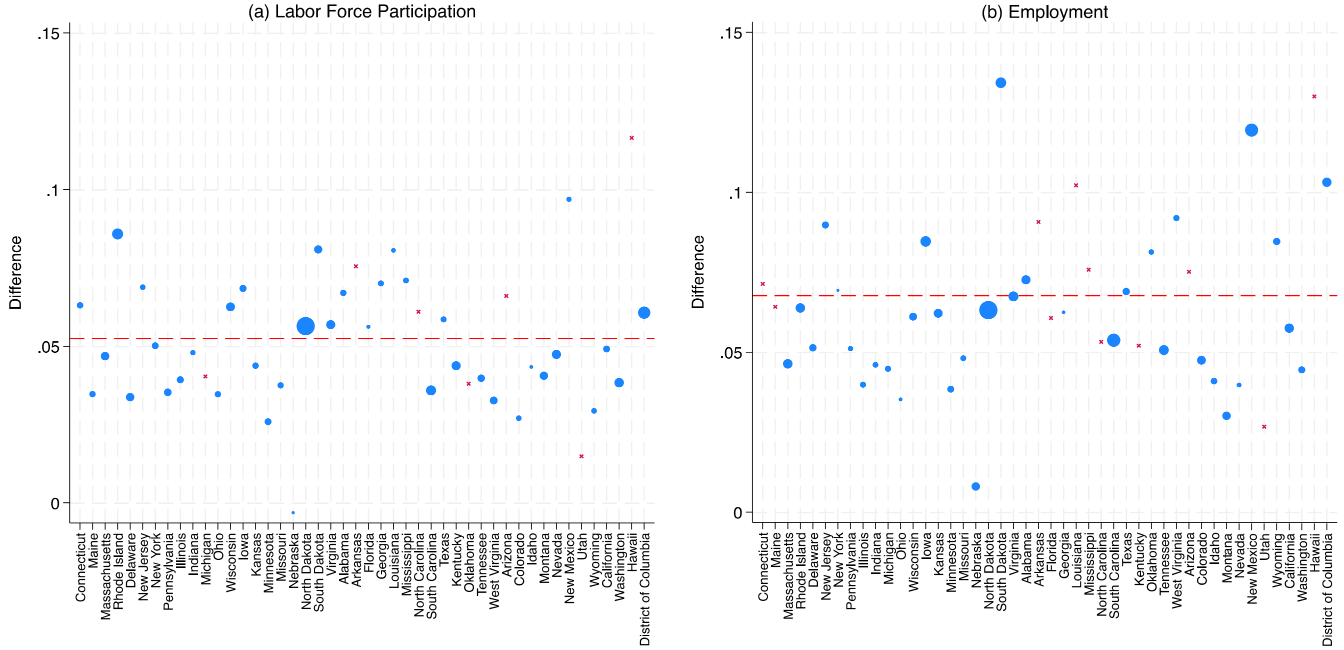

© 2024 Kakara M et al. *JAMA Health Forum*.

eFigure 4. Unit Weights for Synthetic Control in New Hampshire-Specific SDID Analyses: Secondary Outcomes (Graph)

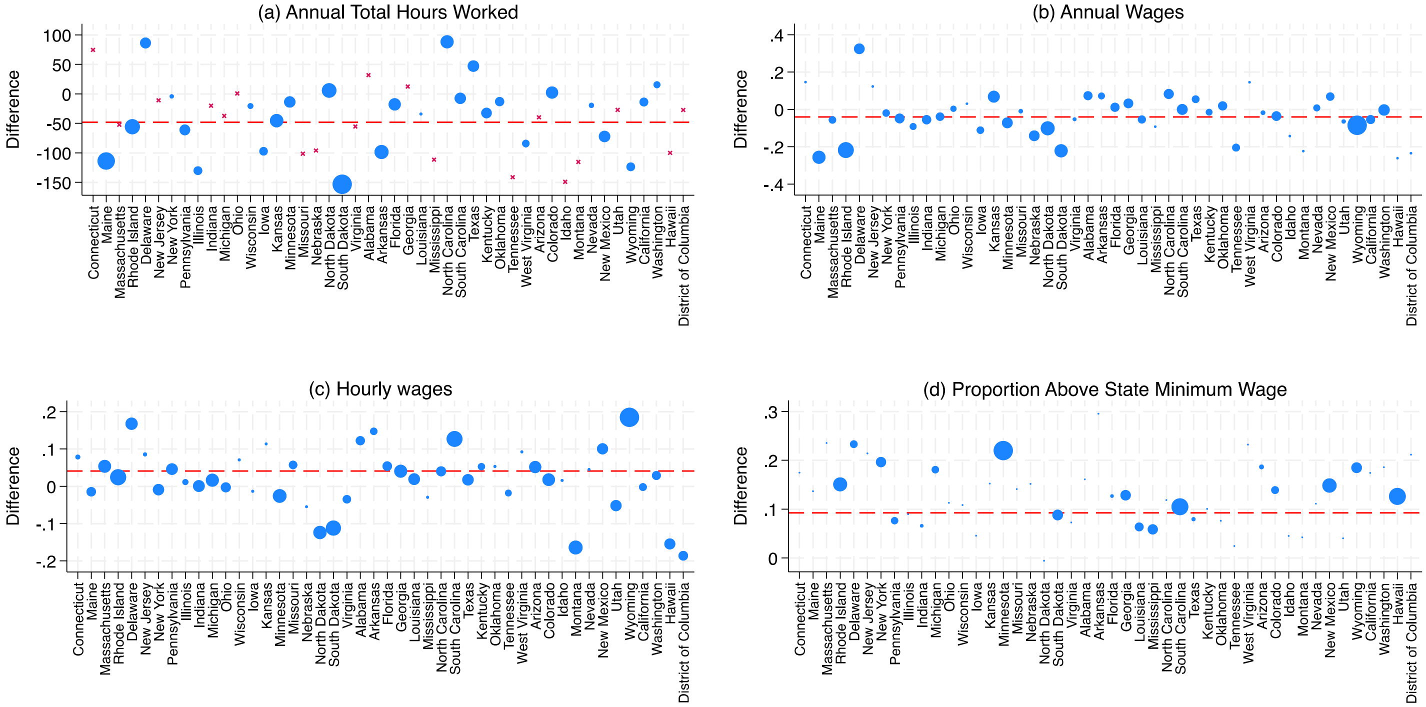

© 2024 Kakara M et al. *JAMA Health Forum*.

eTable 2. Unit Weights for Synthetic Control in Maryland-Specific SDID Analyses

State	Labor Force Participation	Employment	Annual Total Hours Worked	Annual Wages	Hourly Wages	Proportion above minimum wage
Connecticut	.02920495	.00550703	.02216635	.01117547	.01475893	.00224301
Maine	.03238136	.01579923	.02552014	.01660949	.02843178	.01200675
Massachusetts	.01345036	.01704817	.02702806	.03617745	.03792099	.00008388
Rhode Island	0	.01945994	.02135304	.05245381	.00063505	.02905297
Delaware	0	0	.01453792	.00061504	.00284613	.00008388
New Jersey	.01905659	.02771138	.01111175	.01363463	.01612162	.02521607
New York	.02491302	.01809831	.01788187	.03189652	.03822094	.04886129
Pennsylvania	.00338067	.00888281	.0172412	.0183773	.023391	.03987492
Illinois	.0044276	0	.01528657	.00964096	.00122194	.00008388
Indiana	.02513656	.02373459	.02218461	.04433715	.04289366	.02953682
Michigan	.02334635	.02830529	.02424532	.04335084	.0634675	.06312906
Ohio	.0209806	.0321174	.01140838	.01537399	.03187119	.00008388
Wisconsin	.02259266	.01767869	.01053901	.0025451	.00934509	.00008388
Iowa	.0134714	.02928137	.01450314	.00061504	.01277501	.00008388
Kansas	.00006999	.01781065	.04237862	.02698617	.0095633	.00646802
Minnesota	.03898288	.03009579	.02645365	.03213849	.04174169	.09291333
Missouri	.03239418	.03822993	.02439196	.02916925	.02839178	.00008388
Nebraska	.01394811	.03155977	.0424656	.0541651	.00803242	.04829715
North Dakota	0	.02196554	.03496051	.00061504	.00063505	.00008388
South Dakota	.02315138	0	0	.00061504	.06204194	.03004819
Virginia	.05229686	.0501507	.03752557	.03246756	.02694316	.01144396
Alabama	.02118505	.00083854	.02348583	.02737137	.01350368	.00008388
Arkansas	.02488912	.03454656	.00738303	.00061504	.00063505	.00008388
Florida	.02819011	.02276159	.0130009	.01470502	.00807015	.00008388
Georgia	.02417218	.03431291	.04300671	.05405081	.03632993	.04576049
Louisiana	.05414705	.04345965	.042922	.04861228	.01167188	.00124978
Mississippi	.00358418	.00210722	.00426683	.00757935	.02253807	.05848573
North Carolina	.01608397	.01795909	.01790402	.02280324	.0250346	.02014067
South Carolina	.01651656	0	.01428418	.031474	.00656948	.03752584
Texas	.01202898	.00169027	.01329534	.0229121	.04385342	.05144591
Kentucky	.01087899	.01775497	.00425648	.00464864	.00063505	.00008388
Oklahoma	0	0	.02063472	.02797256	.0201586	.04329946
Tennessee	.01190127	.03233605	.03272529	.0260472	.02484653	.0298914
West Virginia	.02836338	.02937369	.02160112	.00061504	.00063505	.00008388
Arizona	.01813916	.03959621	.02087412	.02633843	.01954361	.04304794
Colorado	.01761105	.00776581	0	.00061504	.00380926	.00702439
Idaho	.06923816	.10490754	.01705625	.04468861	.02219702	.00008388
Montana	0	0	.03656027	.01632138	.05634073	.02301541
Nevada	.04278059	.05414183	.0203323	.02061523	.00743236	.00008388
New Mexico	.01772791	.00388731	.02274067	.0050613	.03149958	.05319053
Utah	.05709826	.06228517	.03100585	.03434657	.03412321	.03989552
Wyoming	.08296056	.05587579	.05451103	.04238886	.03077871	.00008388
California	.01422587	.00096322	.02183435	.01597667	.01435187	.0173659
Washington	0	0	.02238688	.02067826	.00162632	.00008388
Hawaii	.0209666	0	.03074854	.00998853	.01128673	.08805969
District of Columbia	.01412546	0	0	.00061504	.05127894	.00008388

© 2024 Kakara M et al. *JAMA Health Forum*.

eFigure 5. Unit Weights for Synthetic Control in Maryland-Specific SDID Analyses: Primary Outcomes (Graph)

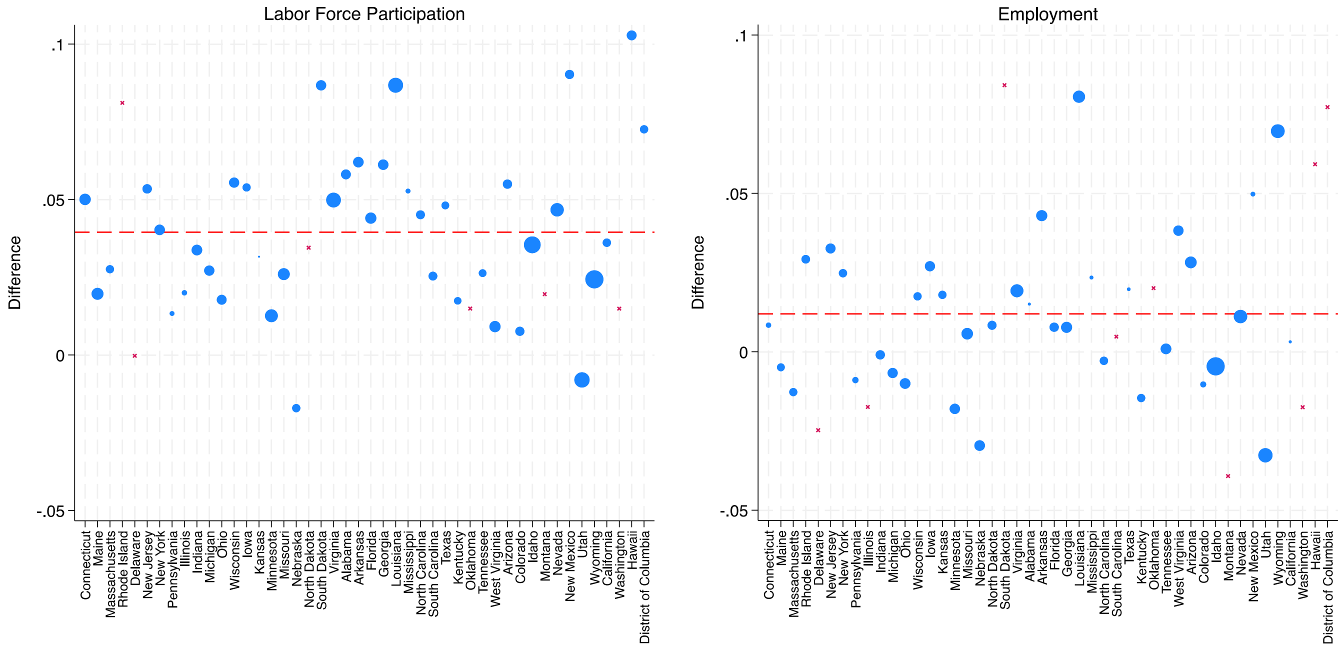

© 2024 Kakara M et al. *JAMA Health Forum*.

eFigure 6. Unit Weights for Synthetic Control in Maryland-Specific SDID Analyses: Secondary Outcomes (Graph)

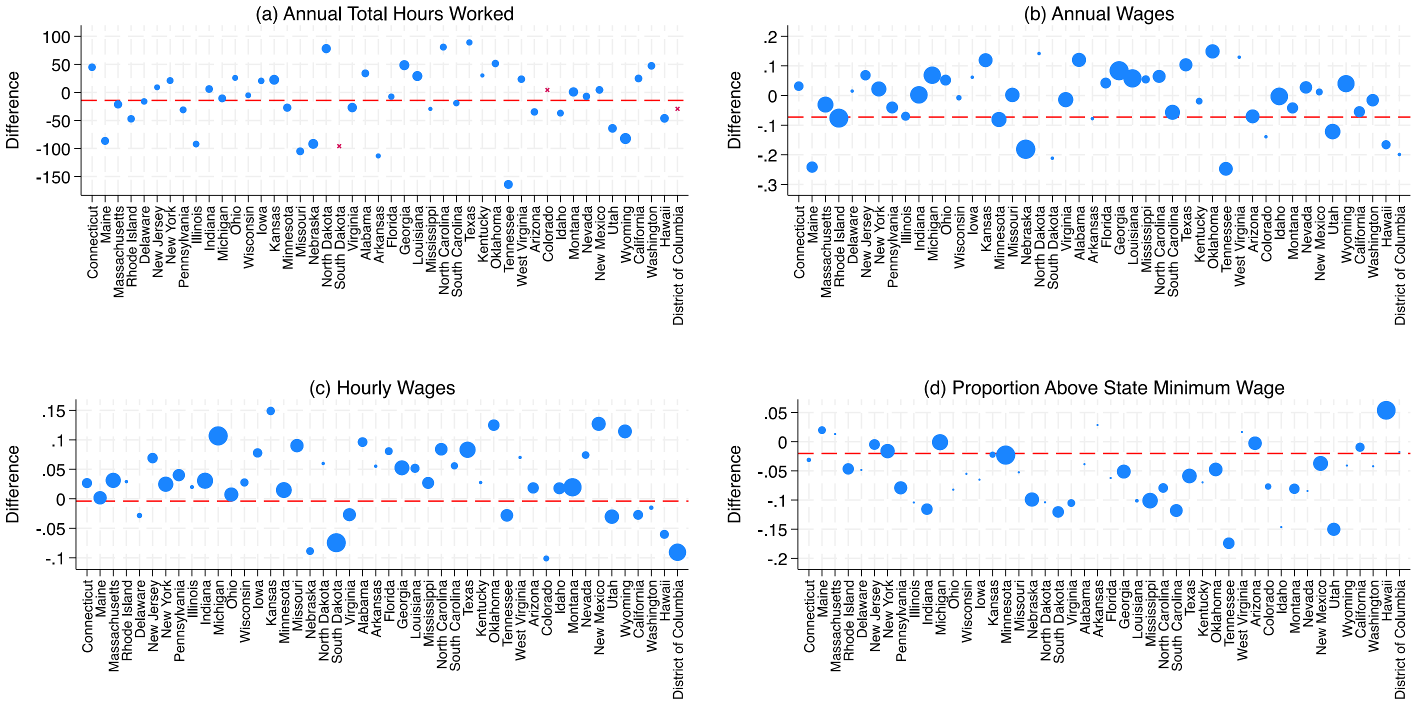

© 2024 Kakara M et al. *JAMA Health Forum*.

eTable 3. Time Weights for Synthetic Control in New Hampshire-Specific SDID Analyses

Year	Labor Force Participation	Employment	Annual Total Hours Worked	Annual Wages	Hourly Wages	Proportion above minimum wage
2010	.31585367	.41660803	.1664295	.32278485	.30888682	.36671413
2011	.11543188	0	.07748268	.07166101	.37696639	.11840694
2012	.2075259	.24075963	.16346894	.27047487	.22082161	.25930341
2013	.20803628	.19934711	.31405526	.05946719	.00216269	.00051734
2014	.15315227	.14328524	.27856362	.27561208	.09116248	.25505818

© 2024 Kakara M et al. *JAMA Health Forum*.

eTable 4. Time Weights for Synthetic Control in Maryland-Specific SDID Analyses

Year	Labor Force Participation	Employment	Annual Total Hours Worked	Annual Wages	Hourly Wages	Proportion above minimum wage
2010	.30446467	.31674837	.11275897	.11940112	.00580632	.09333158
2011	.14274016	0	.10071627	.00171843	.00241454	.01396194
2012	.19919777	.24892973	.11046216	.26985732	.37899029	.24529109
2013	.28651465	.14562827	.32236224	.2091187	.1559291	.00389808
2014	.06708275	.16898461	.12683173	.04729889	.00241454	.08323186
2015	0	.11970902	.22686863	.35260553	.45444521	.56028546

© 2024 Kakara M et al. *JAMA Health Forum*.

eTable 5. Falsification Tests

	Pooled Estimates			New Hampshire			Maryland		
	Beta (95% CI)	Standard Error	p-value	Beta (95% CI)	Standard Error	p-value	Beta (95% CI)	Standard Error	p-value
People with no cognitive disability									
Labor Force Participation	0.00139 (-0.00826, 0.01103)	0.00492	0.78	0.00134 (-0.01209, 0.01476)	0.00685	0.85	0.00394 (-0.00626 0.01413)	0.00520	0.45
Employment	0.00053 (-0.01260,0.01365)	0.00669	0.94	-0.00432 (-0.02444, 0.01581)	0.01027	0.68	0.00648 (-0.00924 0.02220)	0.00802	0.42
People with other (non-cognitive) disabilities[a]									
Labor Force Participation	0.00794 (-0.03451,0.05039)	0.02166	0.71	0.01824 (-0.03412, 0.07059)	0.02671	0.50	-0.00040 (-0.04627, 0.04547)	0.02340	0.99
Employment	-0.02849 (-0.07078, 0.01380)	0.02158	0.19	-0.04604 (-0.10359, 0.01151)	0.02936	0.12	-0.01035 (-0.06483, 0.04412)	0.02779	0.71

[a] Non-cognitive disabilities include the following types of disabilities: vision, hearing, self-care, independent-living and mobility disabilities.

eTable 6. Estimates for New Hampshire, Maryland and Pooled Analyses Using Standard Synthetic Control Methodology

	**New Hampshire**			**Maryland**			**Pooled Estimates**		
	**Beta (95% CI)**	**Standard Error**	**p-value**	**Beta (95% CI)**	**Standard Error**	**p-value**	**Beta (95% CI)**	**Standard Error**	**p-value**
**Primary Outcomes**									
Labor Force Participation[a]	0.065 (0.005, 0.124)	0.030	0.03	0.040 (-0.018, 0.098)	0.02967	0.18	0.054 (0.010, 0.097)	0.022	0.02
Employment[b]	0.086 (0.029, 0.143)	0.029	0.003	0.012 (-0.053, 0.076)	0.03297	0.72	0.053 (0.012, 0.094)	0.021	0.01
**Secondary Outcomes**									
Annual wages[c] (logged)	-0.033 (-0.293, 0.226)	0.13240	0.80	-0.021 (-0.204, 0.162)	0.09349	0.82	-0.028 (-0.20, 0.14)	0.087	0.75
Annual hours worked[d]	-82.52 (-2.40e+02,74.56)	80.143	0.30	-56.74 (-1.78e+02, 64.542)	61.87974	0.36	-71.064 (-1.64e+02, 22.37)	47.67	0.14
Hourly wages[e] (logged)	0.134 (-0.059 0.33)	0.09833	0.17	0.0098 (-0.138, 0.157)	0.075	0.90	0.079 (-0.064, 0.221)	0.0728	0.28
Proportion earning above state minimum wage, %	13.87 (5.17, 22.57)	0.04438	0.002	0.018 (-0.071, 0.107)	0.045	0.69	8.504 (2.805, 14.203)	0.029	0.003

© 2024 Kakara M et al. *JAMA Health Forum*.

eFigure 7. Primary Outcomes for New Hampshire Using Standard Synthetic Control Methodology

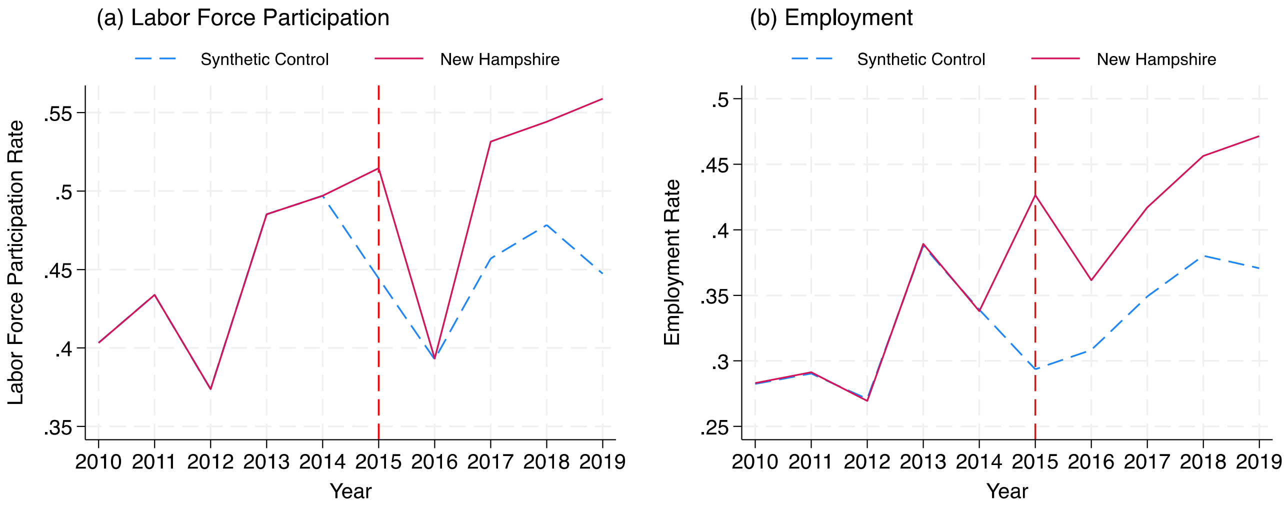

© 2024 Kakara M et al. *JAMA Health Forum*.

eFigure 8. Secondary Outcomes for New Hampshire Using Standard Synthetic Control Methodology

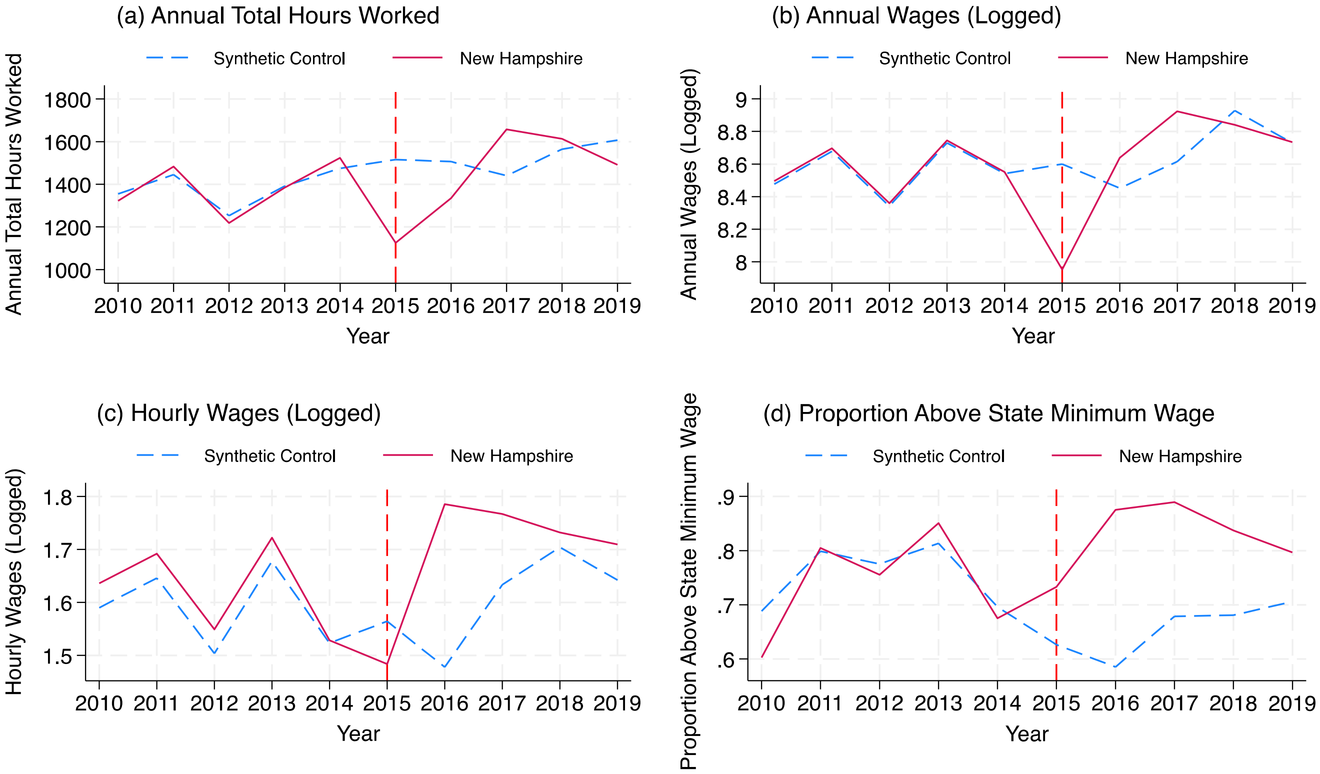

© 2024 Kakara M et al. *JAMA Health Forum*.

eFigure 9. Primary Outcomes for Maryland Using Standard Synthetic Control Methodology

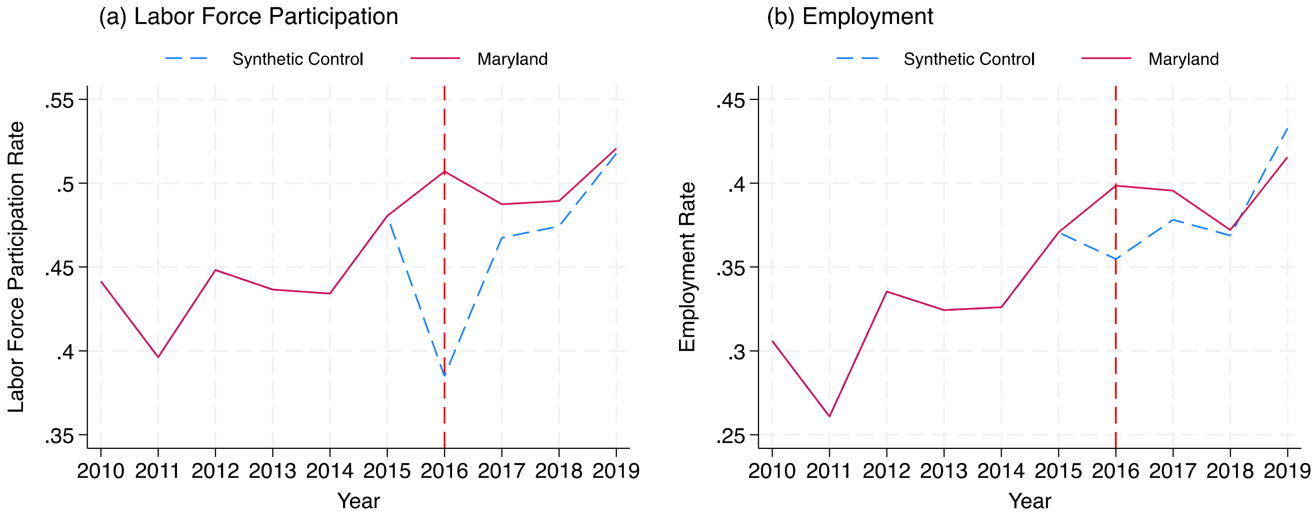

© 2024 Kakara M et al. *JAMA Health Forum*.

eFigure 10. Secondary Outcomes for Maryland Using Standard Synthetic Control Methodology

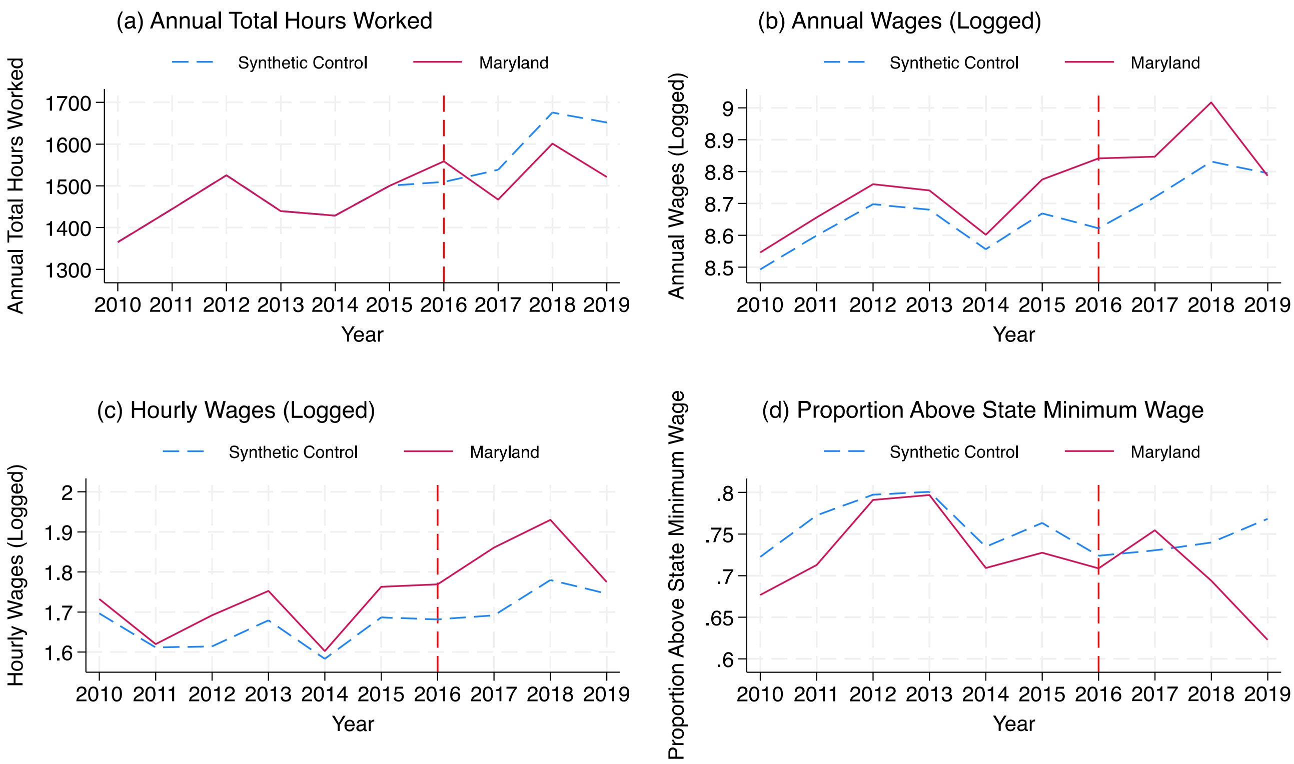

© 2024 Kakara M et al. *JAMA Health Forum*.

eFigure 11. Per-Capita State Funding for Competitive Integrated Employment for Individuals With Intellectual Disabilities

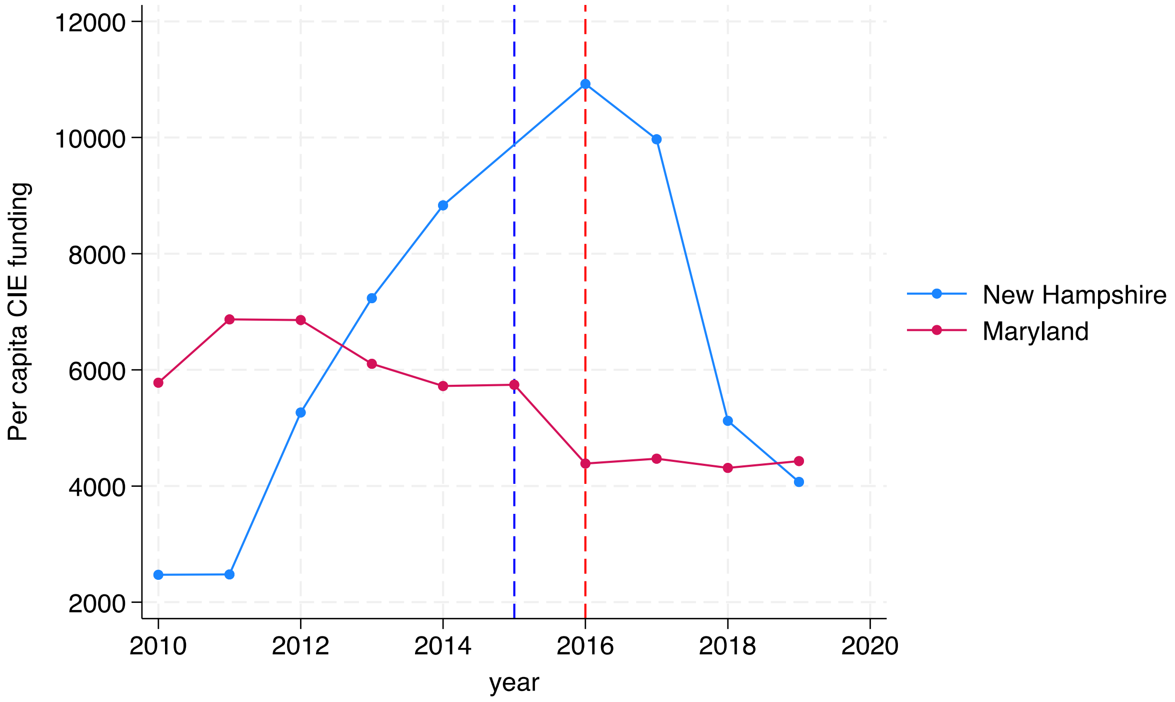

Per capita funding trends for competitive integrated employment (CIE) for individuals with intellectual disabilities from 2010-2019 for New Hampshire and Maryland. Dashed lines indicate the year in which Section 14(c) was repealed for the respective states. Data obtained from ThinkWork, an online data hub that includes data from the National Survey of State IDD Agencies' Employment and Day Services. Per-capita funding was calculated by divided total funding for integrated employment divded by number of people with IDD served.

© 2024 Kakara M et al. *JAMA Health Forum*.
