# Supplementary material for: Repeal of Subminimum Wages and Social Determinants of Health Among People With Disabilities
Source: JAMA Health Forum. 2024 Nov 15;5(11):e244034. doi: 10.1001/jamahealthforum.2024.4034 (PMC11568457; doi:10.1001/jamahealthforum.2024.4034)
Supplement: Supplement 2. — Data Sharing Statement [file jamahealthforum-e244034-s002.pdf]

Data Sharing Statement

Kakara. Repeal of Subminimum Wages and Social Determinants of Health Among People With Disabilities. *JAMA Health Forum*. Published November 15, 2024.
doi:10.1001/jamahealthforum.2024.4034

Data
Data available: No

Additional Information
Explanation for why data not available: Data used in this study is publicly available through the U.S. Census Bureau, and ipums.org
